# AIDS fighter health defense: protocol for a randomized controlled trial to test a game-based intervention to improve adolescents’ AIDS prevention ability

**DOI:** 10.1186/s12879-021-06161-0

**Published:** 2021-05-22

**Authors:** Jian Tang, Yanhua Chen, Xingli Yu, Jianlan Ren, Mei Li, Yue Luo, Hong Xie, Jing Wen

**Affiliations:** 1 Department of Central Sterile Supply, the Affiliated Hospital of Southwest Medical University, 25 Taiping Street, Luzhou, China; 2 Department of Nursing, the Affiliated Hospital of Southwest Medical University, 25, Taiping Street, Luzhou, China; 3 Southwest Medical University, School of Nursing, 1 Xianglin Road, Luzhou, China; 4 Department of Operating Room, the Affiliated Hospital of Southwest Medical University, 25 Taiping Street, Luzhou, China

**Keywords:** Adolescents, AIDS prevention ability, Game-based intervention, Information-motivation-behavioral skills model

## Abstract

**Background:**

Although great progress has been made in the prevention and treatment of AIDS, there are still a considerable number of new infections annually, especially in adolescents. With the advance of technology, game-based education has gradually become an important tool for changing healthy behaviors among youth.

**Methods:**

A protocol for conducting a randomized controlled trial to evaluate the “AIDS Fighter · Health Defense”, a game-based AIDS education project aimed at improving the ability of adolescents to prevent AIDS. During the four-week intervention, participants will receive: 1) A virus combat game; 2) Goal setting to eliminate HIV; 3) Questions to be answered to be resurrected in the game; 4) Points ranking; 5) Recognition and Rewards. The primary outcomes include changes in participants’ knowledge, stigma attitude, and risk behaviors attitude related to AIDS after four weeks of intervention. The secondary outcomes are the participants’ AIDS-related risk behaviors three and six months after the intervention.

**Discussion:**

AIDS Fighter· Health Defense may be an innovative approach to help adolescents improve AIDS prevention capabilities, fill the gap in game-based AIDS prevention education in China, and gain experience of AIDS management.

**Trial registration:**

Chinese Clinical Trial Registry: ChiCTR2000040195. Registered 25 November 2020.

## Background

Acquired Immunodeficiency Syndrome (AIDS) is a global disease that seriously threatens human health [1]. By the end of 2019, 38 million people worldwide are living with human immunodeficiency virus (HIV) [2]. At the end of October 2019, China reported 958,000 survivors of HIV infection [3], and the number of newly diagnosed HIV infection cases among adolescents aged 15–24 each year is approximately 3000 in recent years [4],which implies that the situation of AIDS prevention is still grim.

Developing countries, represented by China, have problems in AIDS prevention education, such as outdated education model, poor effect of education, and uneven distribution of educational resources [5, 6]. Therefore, it is necessary to develop innovative AIDS education. With the development of science and technology, AIDS prevention education has changed from traditional methods to modern methods. Among them, game-based intervention has become a significant way to change healthy behaviors [7–9]. In recent years, some randomized controlled trials conducted in the United States and Africa targeting 11–15 years old adolescents have found that game-based AIDS education could improve knowledge, attitudes and behaviors related to AIDS, and increase the effectiveness of AIDS prevention education, additionally, adolescents prefer game-based AIDS education, and are more willing to recommend it to their friends [10–13]. However, there are few games-based HIV education intervention studies in China, especially randomized controlled trials to evaluate the effects of game education.

Below we describe the protocol of our randomized controlled trial to evaluate a game-based intervention to improve adolescents’ AIDS prevention ability in China. The intervention, called AIDS Fighter · Health Defense, is a 4-week game experience, and includes components designed to increase AIDS-related knowledge, improve AIDS prevention motivation, and strengthen AIDS prevention behaviors among adolescents. This study is guided by the Information-Motivation-Behavioral Skills Model (IMB Skills Model), which believes that the prevention of AIDS requires comprehensive intervention from three aspects: AIDS-related information, motivation, and behavior skills [14]. In the AIDS Fighter · Health Defense intervention, adolescents will actively or passively receive AIDS prevention information, repeatedly train AIDS prevention skills, and strengthen the motivation to prevent AIDS. The combination of this study and theory is shown in Table 1.

**Table 1:** The combination of AIDS Fighter · Health Defense and IMB Skills Model

IMB Skills Model	AIDS Fighter · Health Defense Intervention
Information	1) By simulating the “clinical manifestations” of the human body after being infected with HIV, users can understand the disease manifestations after being infected with HIV. 2) Through the “knowledge corner” and “answer questions” during the battle, the information on AIDS-related knowledge is passed to the players.
Motivation	1) The goal of this game is to eliminate HIV, which can strengthen players’ motivation to eliminate the virus. 2) The “points ranking” in the game can stimulate players’ motivation to use this game. 3) “Knowledge corner” can help players acquire AIDS-related knowledge and further stimulate healthy players to have the motivation to prevent HIV infection.
Behavioral skills	1) The behavior training of using condoms, improving antiviral medicine use, avoiding alcohol and drugs has been added to strengthen the player’s refusal of dangerous sex, refusal of intravenous drug use, and obtain antiretroviral medicine for pre-exposure prophylaxis (PrEP)

## Methods/design

### Study design

**Fig. 1:**
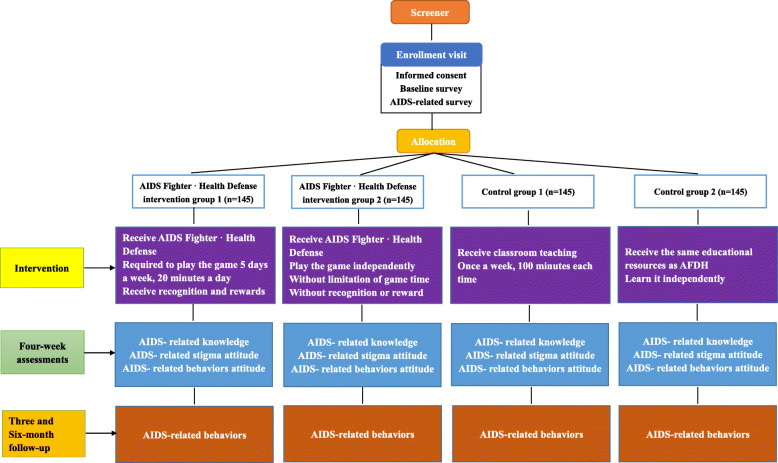
Study workflow diagram

Study participants will be recruited from adolescent volunteers and allocated to one of the four randomized groups after completing a baseline survey (Fig. 1) (AIDS Fighter · Health Defense intervention group 1; AIDS Fighter · Health Defense intervention group 2; control group 1; control group 2). Participants are required to complete the assessment four weeks later and three-month, six-month follow up visit after the intervention. Our hypotheses are:

1. There is no statistical difference between AIDS Fighter · Health Defense intervention group 1 and AIDS Fighter · Health Defense intervention group 2 in AIDS-related knowledge, stigma attitude, risk behaviors attitude and the incidence of risk behavior;
2. Compared with adolescents in the control groups, those in the AIDS Fighter · Health Defense intervention groups will have More AIDS-related knowledge, less AIDS-related stigma and fewer AIDS-related risk behaviors.
3. Comparison between control group 1 and control group 2, group 1 will show better effects in improving AIDS-related knowledge, attitude and behaviors.

### Study participants

This study is aimed at improving the AIDS prevention capabilities of adolescent students. The inclusion criteria are: 1) Students aged 15–24 years; 2) Participants have clear consciousness, no mental disorders, and able to read and express themselves; 3) Participants were informed about the study and voluntarily participated in the study (< 16 years old requires the consent of the guardian). Those who are diagnosed as HIV positive will be excluded from the study.

### AIDS fighter · health defense: overview

#### Game design

Goal setting has been identified as an effective behavior change procedure [15]. The storyline of the game is that HIV launched an attack on the human body, and the goal is to control the hero to fight with HIV and eliminate it. During the battle, the hero must obtain antiretroviral medicine in the game and refuse the behaviors of condomless sex, drugs and alcohol.

**Fig. 2:**
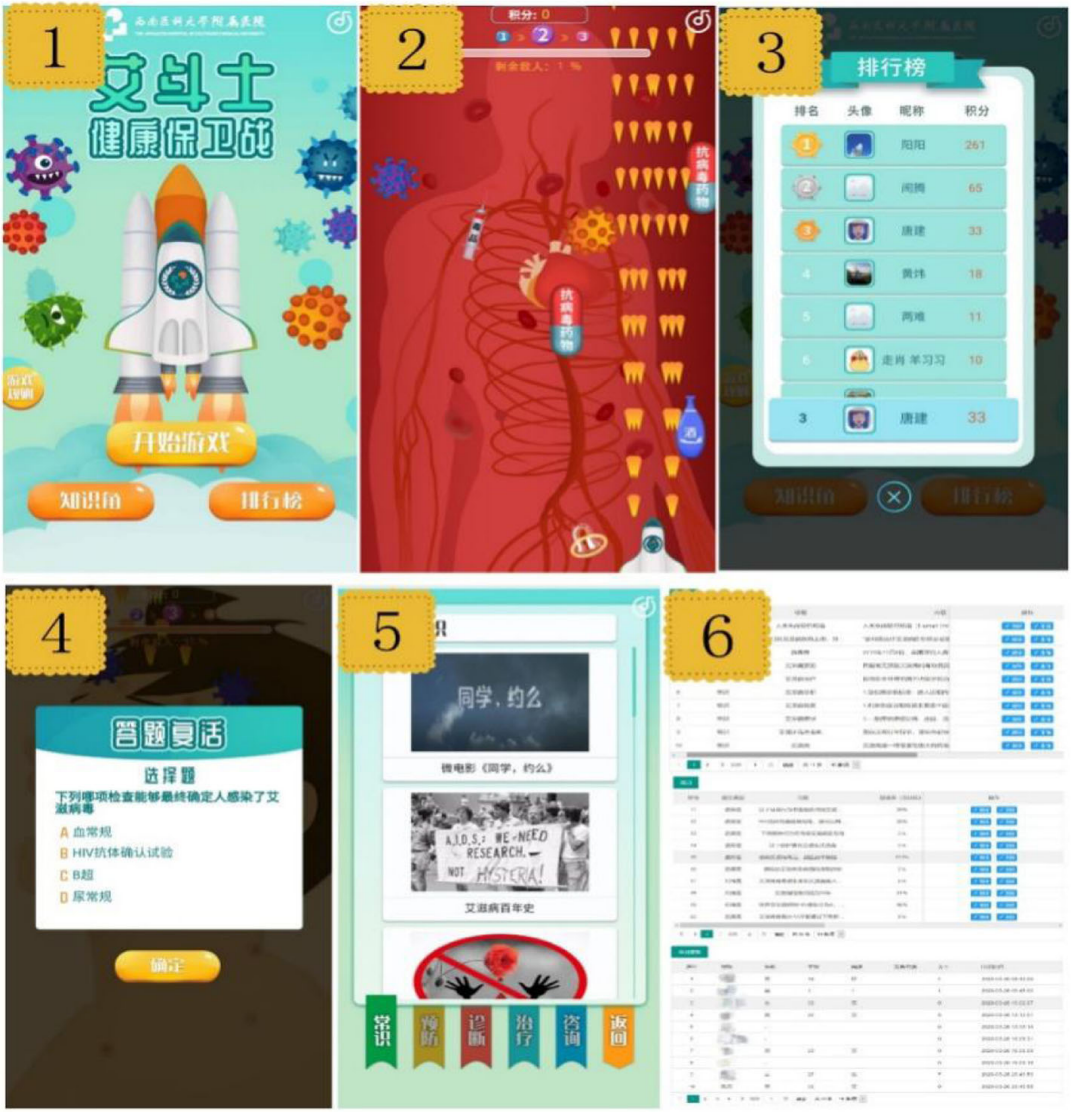
Game design. (There are 6 interfaces in the picture. *Interface 1* is the login interface, including buttons of “start game”, “knowledge corner” and “point rankings”; *Interface 2* is the battle process of the game; *Interface 3* is a ranking of points; *Interface 4* is the “Challenge to answer quizzes”; *Interface 5* is the “knowledge corner”; *Interface 6* is the data management system)

Seven levels were contained in the game: immune system, blood system, skin and mucous membrane system, nervous system, respiratory system, digestive system, urogenital system. There will be corresponding animation prompts for the success or failure of the game. If they fail, it will prompt them that HIV has invaded the human body, and display pictures of symptoms corresponding to the level of content as a warning education. The game interface is shown in Fig. 2.

#### Challenge to answer quizzes

Participants will have a chance to answer questions if they failed during the battle. If they answer correctly, they will be resurrected and continue to fight, otherwise, the game will fail. Questions include knowledge of AIDS prevention, antiretroviral treatment, pre-exposure and prophylaxis.

#### Knowledge corner

The “knowledge corner” contains a variety of AIDS knowledge, as AIDS prevention, diagnosis, antiretroviral treatment, pre-exposure prophylaxis, etc. What is taught in the form of texts, pictures and videos. Players can earn points by reading articles or watching videos, and the points are included in the rankings; Moreover, players can leave a message in the “Consultation” column, or conduct online consultation by identifying the QR code of the Internet hospital.

#### Points and rankings

Points could be earned by logging in to the game, breaking through levels, reading articles or watching videos, etc. The point for logging in is 1, once a day. Points for breaking through levels is 5 for the first level, 10 for the second level, 15 for the third level, and so on, and may only be accumulated once a day. Two points can be awarded for reading an article or watching a video for more than 30 s. One point could be earned each time if they pick up a condom or antiviral medicine during the game, and the points can be accumulated. The points and rankings of themselves and their friends in the game can be viewed.

#### Game data management

Educational resources can be edited and upload to the “knowledge corner” through the management system, and the system can realize the following data collection functions:1) Player’s basic personal information; 2) Player’s points and rankings; 3) Player’s message and consultation information; 4) Player’s answers to questions; 5) When did the player log in to the game and how long has it been used; and 6) What did the players learn in the “knowledge corner”.

### Intervention group: overview

#### AIDS fighter · health defense intervention group 1

Participants will receive the educational game “AIDS Fighter · Health Defense” and will be required to play the game and learn AIDS education essays or videos in the “knowledge corner” at least 5 days a week and 20 min a day. A data management system will be used to monitor the time participants played in the game, and whether they have learned the content in the “knowledge corner”. After four weeks of intervention, the effect of the intervention will be evaluated by using the same questionnaire as applied in the baseline survey.

Participants will be informed that they will be recognized and rewarded after completing daily game tasks and completing four-week follow-up assessments according to the points ranking. The top 20% will receive a “Gold Award” and 50 RMB in cash, the middle 30% will receive a “Silver award” and 50 RMB in cash, and the lowest 50% will receive a “Bronze award” and 20 RMB in cash.

#### AIDS fighter · health defense intervention group 2

Participants will receive “AIDS Fighter · Health Defense”, without limitation in time to play the game or learn the content of the “knowledge corner”, and there are no incentives as recognition or rewards. Participants play this game according to their preference. Four weeks later, the same questionnaires will be used to measure the effect of their use of the game.

### Control group: overview

#### Control group 1

Participants will receive classroom teaching once a week for 100 min each time. The total time is the same as the time required by the AIDS Fighter · Health Defense intervention group 1. A regular teacher will conduct AIDS prevention classroom education on a fixed day each week and the content of the lecture is consistent with the knowledge in the game.

#### Control group 2

Participants will be added to the QQ chat group (one of the most popular social tools in China), and the same educational resources as the “knowledge corner” in AIDS Fighter · Health Defense will be uploaded to the QQ group. Participants will then be reminded to learn the knowledge by themselves daily. But there are no gamification elements, such as barriers, points ranking, answering questions, rewards, etc.

### Recruitment, randomization, and allocation concealment

#### Recruitment

Participants will be recruited through online recruitment and on-site recruitment. The sufficient statistical power to detect changes in AIDS knowledge, attitude and behaviors after the intervention are considered in sample size calculation, and131 participants are need in each group to maintain 80% statistical power. Assuming the attrition rate is 10%, the sample size of each group is determined to be 145, and the total sample size is 580.

The study site is Luzhou City, Sichuan Province, China. Participants will be recruited from secondary school, high school, college and university. When they agree to participate in the research and complete the follow-up measurement and informed consent form will need to be signed for confirmation. After completing the research, each participant will receive a reward of 30 RMB in cash.

#### Randomization and allocation concealment

Opaque envelopes will be prepared for randomization. In 580 envelopes, each will contain a note with the words “ AIDS Fighter · Health Defense Intervention Group 1” or “ AIDS Fighter · Health Defense Intervention Group 2” or “Control Group 1” or “Control Group 2” (145 envelopes per group). Participants will be randomized to select an envelope, then assigned to the corresponding group according to the information on the envelope. The data analysts are blinded to the allocation.

### Outcomes and data collection

The primary outcomes are AIDS-related knowledge, AIDS-related stigma attitude and AIDS-related behaviors attitude before and four weeks after the intervention. AIDS-related knowledge will be assessed through a self-made questionnaire with a total of 45 items, one point for a correct answer. The higher the score, means the more knowledge participants have learned. AIDS-related behaviors attitude will be assessed through a questionnaire with eight items, with one point for a correct answer. The higher the score, means the lower the risk of AIDS-related behaviors among participants. Five experts were invited to verify the content validity of these questionnaires, the item-level CVI (I-CVI) and the scale-level CVI (S-CVI) are both 1. 00. AIDS-related stigma attitude will be assessed through the Chinese version of Zelaya’s HIV/AIDS Stigma Scale, which was compiled by Zelaya [16], with 24 items in total, and was Sinicized by Xing Haiyan [17]. After Sinicization, the I-CVI was 0. 82 ~ 1. 00, and the S-CVI was 0. 97. Each item uses a score of 1–5. The higher the score, the more the participants’ stigma of AIDS.

Secondary outcomes are the AIDS-related behaviors followed up for three and six months after the intervention. Participants will receive cash rewards after completing the evaluation. AIDS-related behaviors will be assessed through the Questionnaire on the Health of Young Students that was issued by the Chinese Center for Disease Control and Prevention, which has 33 items and is the official questionnaire for the investigation of AIDS-related behaviors among adolescents in China. The fewer AIDS-related risk behaviors, the more the participants have gained AIDS prevention capabilities.

### Data management and statistical analysis

All data will be directly recorded in the online database (www.wjx.cn, Ranxing information technology co., Ltd., Changsha, China). The database system automatically saves the data and ensures the safety, integrity and consistency of the data. The web server is hosted in Alibaba Cloud (Alibaba Group, Hangzhou, China) and is protected by a corporate firewall, while a daily backup mechanism can ensure data security. Data has multi-level permission settings and password protection to ensure data security. In statistical analysis, general statistical analysis will be carried out by using standard statistical software and professional statistical software.

Analysis of Variance (ANOVA) will be used to find out whether there have differences between the AIDS Fighter · Health Defense intervention groups and the control groups in AIDS-related knowledge scores and attitude scores after the intervention of four weeks. Repeated Measures ANOVA will be used to see whether there have differences on the incidence of AIDS-related behaviors followed up for three and six months after the intervention in four arms. Intention-to-treat analysis will be used to account for the influence of attrition. The difference was statistically significant when *P* ≤ 0. 05.

### Ethical approval and informed consent

This study has been approved by the Ethics Committee of Clinical Trials, Affiliated Hospital of Southwest Medical University, with the approved number: KY2020001. Participants who sign up will be screened, for those who meet the inclusion criteria, we will issue informed consent to them, and inform them in detail of the study purpose and process, number of participants, study duration, potential risks and benefits, random allocation, and data confidentiality.

## Discussion

In developed countries, the focus of AIDS prevention education is on the sexual behavior of high-risk groups, but the focus of AIDS education in our study is on the AIDS-related knowledge of adolescents. The awareness rate of AIDS knowledge among the public in developed countries might be higher than China, and existing studies are mainly aimed at high-risk groups, such as men who have sex with men and adolescents who have sex without condoms [18, 19]. But in China, the awareness rate of AIDS among the public is low [20], especially adolescents in poor areas and western China [21, 22].

Adolescents are in the sexually active period are a high-risk group of HIV infection [23], the awareness rate of AIDS among them is low [24], and the HIV infection rate is high in China [25], AIDS preventive education for them is crucial. Therefore, the target of HIV prevention education in China should be the public, especially adolescents.

To meet the need of AIDS education of adolescents, the educational game “AIDS Fighter · Health Defense” was developed. As mentioned above, AIDS Fighter · Health Defense is a theory-based AIDS education game that can provide knowledge and skills to adolescents to prevent AIDS. Whether adolescents are interested in AIDS Fighter · Health Defense and insist on using it is the key to this study. If the game does not appeal to adolescents, it will not be popularized among them, nor have educational significance. To promote the use of this game by adolescents, the game is designed to be highly interactive and interesting, including gamification elements as levels, points, answering questions, rankings, and rewards. In the levels of the game, seven different organs and systems of the human body are simulated, it can vividly show the way HIV invades the human body and its performance if infected with HIV. This can not only improve the fun of the game, but also popularize the process of HIV invading the human body, which may increase users’ willingness to play the game and prevent AIDS.

Four arms were designed in our research, including two intervention arms and two control arms. Participants in the two intervention groups will receive AIDS Fighter · Health Defense, one of which will strictly control the time spent in the game and provides incentives to increase motivation to use the game; Another group will receive AIDS Fighter · Health Defense, but without time limitation or incentives; One of the two control groups is classroom teaching and the other is autonomous learning. By comparing the intervention groups with the control groups, it can be found whether AIDS Fighter · Health Defense is better than classroom teaching and autonomous learning in improving participants’ AIDS prevention ability; By comparing the two intervention groups, we can figure out whether AIDS Fighter · Health Defense is attractive, whether players will voluntarily use the game to improve AIDS prevention capabilities. This will determine whether the game can be promoted on a large scale. By comparing the two control groups, we can understand the difference between classroom learning and autonomous learning in improving AIDS prevention capabilities. Such a design could see the differences in the effects of game-based intervention, classroom teaching, and independent learning in AIDS education. And provide evidence for choosing different AIDS education methods.

We hope that the game-based interventions can be widely used in AIDS education in China and other countries. However, some countries are exam-oriented in evaluating the effect of education and there may be some resistance to the promotion and application of the game; parents may worry that their children will be addicted to the game and affect their academic performance [26, 27]. To address this problem, we can perform identity authentication when the player enters the game and prevent minors from being addicted to the game by limiting the online time.

“AIDS Fighter · Health Defense” could be suitable for the public, high-risk groups of AIDS, and HIV-infected people. It could be an auxiliary educational tool for AIDS prevention for the public and high-risk groups, and for HIV-infected people to maintain long-term ART adherence and keep virus suppression. We believe that with the development of this study, we can provide an AIDS education method for China and other countries to improve the ability to prevent AIDS of public and the quality of life of HIV infected.

## Data Availability

The data collected in this study will be published in papers after the study is completed. And the original data will be uploaded to the Chinese Clinical Trial Registration Center, which can be found by searching for the registration number of this study.

## Abbreviations

AIDS: Acquired Immune Deficiency Syndrome
HIV: Human Immunodeficiency Virus
IMB Skills Model: Information-Motivation-Behavioral skills model
PrEP: Pre-exposure Prophylaxis
CVI: Content Validity Index
I-CVI: the Item-level CVI
S-CVI: the Scale-level CVI
ANOVA: Analysis of Variance
